# Synthesis of metallo-supramolecular materials based on terpyridine functionalized double-decker silsesquioxane with improved complexation efficiency

**DOI:** 10.3906/kim-1909-60

**Published:** 2020-04-01

**Authors:** Asuman ÇELİK KÜÇÜK, Jun MATSUI, Tokuji MIYASHITA

**Affiliations:** 1 Department of Metallurgical and Materials Engineering, Marmara University, İstanbul Turkey; 2 Department of Material and Biological Chemistry, Yamagata University, Yamagata Japan; 3 Institute for Multidisciplinary Research for Advanced Materials, Tohoku University, Sendai Japan

**Keywords:** Double-decker silsesquioxane, ruthenium(II)-terpyridine complex, complexation procedures

## Abstract

Silsesquioxane-based transition-metal complexes have come to the forefront due to the ability of silsesquioxane to control nanostructures and properties. However, some difficulties in complete complexation and purification limit the widespread use of transition-metal-based supramolecular coordination complexes comprising silsesquioxane. Herein, 2 different approaches have been proposed for the synthesis of metallo-supramolecular materials on the basis of ruthenium(II)-terpyridine functional double-layer silsesquioxane (DDSQ) (Tpy/Ru-DDSQ) (Routes 1 and 2). In Route 1, complexation was followed by functionalization of DDSQ with the ligand, whereas in Route 2, complexation was performed before the ligand was inserted into the DDSQ. Tpy/Ru-DDSQ obtained from both approaches was characterized by ^1^H NMR, X-ray photoelectron spectrometer, and FTIR and found in the same structure. Both methods were fully discussed and their merits were explored. The complexation yield of the routes was similar. However, the results based on NMR and UV-Vis spectroscopy demonstrated that the incorporation rate of DDSQ into the complex was quite high in Route 2. As far as is known, this is the first study based on the effects of complexing Tpy ligands before/after binding to the target compound, particularly to silsesquioxane-based materials.

## 1. Introduction

It has long been known that stable transition-metal complexes play a significant role in increasing the durability and efficiency of light-to-electricity conversion systems [1], electroluminescent systems [2], organic light-emitting electrochemical cells [3], and nonlinear optical devices [4,5]. In this respect, polypyridine chromophores as chelating ligands are preferred owing to their ability to form very stable transition-metal complexes. On the other hand, it is well known that polymer frameworks with high thermal and chemical stability play important roles in obtaining more stable metallo-supramolecules and also indirectly contribute to the abovementioned application efficiency [6,7].

Polyhedral oligomeric silsesquioxane (POSS)-based materials consist of a highly rigid and stable inorganic SiO1.5 core [8–12] in addition to readily functionalized organic coronae [11,13–16]. They can therefore be used to obtain stable transition-metal complexes. However, it is well known that the synthesis of POSS-based transitionmetal complexes is difficult and does not have practical significance due to low yield and partial complexation. On the other hand, if complete complexation of POSS is achieved, this would open the door to using highly stable POSS-based metallo-supramolecules for a wide range of applications. Therefore, there has recently been growing interest in the use of POSS derivatives as part of the transition-metal-based supramolecular coordination complex. For example, POSS-functionalized platinum(II) terpyridine (Tpy) complexes were demonstrated to exhibit drastic morphological transformation with the changing of the solvent medium [17]. In another study, a coordination polymer complex based on POSS with a stable Tpy-metal complex was developed [18]. In addition, a highly functionalized molecular system incorporating a POSS derivative as the central core of a dendrimer was introduced [19]. Finally, a double-decker silsesquioxane (DDSQ)-based bis(terpyridine) ruthenium(II) (Ru(II)) complex was synthesized and its anodic photocurrent response was investigated. The efficient anodic photocurrent response was attributed to the perfect arrangement of Ru(II)-bis(terpyridine) moieties into the DDSQ nano building blocks [20]. In all of the aforementioned studies, complexations were performed after functionalizing of the silsesquioxanes. However, the complexation effect of the chelating ligands before attachment of the target compound has not been discussed to date.

In this presented work, 2 different approaches (Routes 1 and 2) were investigated to synthesize metallosupramolecular materials based on Ru(II)-Tpy-functionalized DDSQ (Tpy/Ru-DDSQ). In the first approach (Route 1), complexation came after functionalization of DDSQ with the Tpy ligand, while in the second approach (Route 2), complexation of Tpy with the Ru(II) metal was performed before Tpy was inserted into the DDSQ core. Both methods are fully discussed and their merits are explored.

## 2. Experimental

### 2.1. Materials

DDSQ containing 2 hydrogen groups (2H-DDSQ) was kindly donated by CNC Corp. (Singapore). Toluene, dimethyl sulfoxide (DMSO), and acetonitrile were purchased from Nacalai Tesque Inc. (Kyoto, Japan) and used without further purification. Platinum divinyl-tetramethyldisiloxane (Pt(dvs), 3 wt.% in xylene solution) was obtained from Umicore (Brussels, Belgium). Diethylene glycol, dichloromethane, chloroform, hexane, acetone, methanol (MeOH), and ethyl ether were purchased from Kanto Chemical Co. Inc. (Tokyo, Japan) and distilled before use. Spectroscopic-grade chloroform (Dojindo Laboratories) was used as a casting solvent. Anhydrous tetrahydrofuran (THF), RuCl_3_ .3H_2_O, 4’-chloro-2,2’:6’,2”-terpyridine (Tpy-Cl), 2,2’:6’,2”-terpyridine (Tpy), and 5-isocyanato-1-(isocyanatomethyl)-1,3,3-trimethylcyclohexane (isophorone diisocyanate) were purchased from Sigma-Aldrich (St. Louis, MO, USA) and used as received.

### 2.2. General methods

FTIR spectra were obtained using a Jasco FTIR 4200 spectrometer (Tokyo, Japan). IR spectra of the DDSQbased materials were recorded between 4000 and 750 cm^-1^ with 4 cm^-1^ resolution under a continuous nitrogen purge. ^1^H NMR measurements were performed with a JEOL JNM-AL 400 spectrometer (Tokyo, Japan) in CDCl_3_ or DMSO-d6 without tetramethylsilane. A PerkinElmer PHI 5600 X-ray photoelectron spectrometer (XPS) (Waltham, MA, USA) was used to control the elemental composition and oxidation state of the elements at the surface. All binding energies in the XPS measurements were referenced to the C 1s peak for neutral carbon, which was assigned a value of 285.0 eV. The takeoff angle was fixed at 45°. UV-Vis absorption spectra were measured using a Hitachi U-3000 UV-Vis absorption spectrometer (Tokyo, Japan).

### 2.3. Synthesis of DDSQ-based Ru(II)-Tpy complex through Route 1

#### 2.3.1. Synthesis of amine functional terpyridine compound, NH_2_ -Tpy

6-Aminohexyl 4’-(2,2’:6’,2”-terpyridinyl) ether (NH_2_ -Tpy) was prepared according to procedures in the literature [19,20]. Briefly, to a stirred suspension of powdered KOH in dry DMSO at 30 °C, 6-amino-hexan-1-ol was added. After 30 min, 4’-chloro-2,2’:6’,2”-terpyridine (Tpy-Cl) was added, and then the mixture was stirred for 4 h at 60 °C and precipitated dropwise into 1.5 L of ice-cooled, distilled water. After 2 h of stirring, the precipitate was collected, washed with distilled water, and dried in a vacuum overnight. The pure product was obtained in a 95% yield. The characterization results obtained were fully consistent with a previous study [20].

Tpy-NH_2_ : ^1^H NMR (ppm) (CDCl_3_ , 400 MHz): δH 8.69 (2H, d, Tpy6–6”), 8.60 (2H, d, Tpy3,3”), 8.00 (2H, s, Tpy3’,5’), 7.84 (2H, t, Tpy4,4”), 7.36 (2H, t, Tpy5,5”), 4.20 (2H,–CH_2_O–), 2.60 (2H, NH_2_ CH_2_ –), 1.85 (2H, –CH_2_ CH_2_O–), and 1.50 (6H, –CH_2_ CH_2_ CH_2_ –).

#### 2.3.2. Functionalizing of DDSQ with terpyridine, Tpy-DDSQ

An excess amount of isophorone diisocyanate (0.71 g, 3.2 mmol) was mixed with NH_2_ -Tpy (1 g, 2.88 mmol) in DMF (50 mL) at 45 °C under an argon atmosphere for 4 h. The progress of the reaction was followed by FTIR. The NH_2_ -Tpy consisted of a functional primary amine group. After the reaction between NH_2_ -Tpy and isophorone diisocyanate, all primary amine was converted to secondary amine. This implied that 2 bands of the asymmetrical N–H stretch and the symmetrical N–H stretch at 3400–3300 cm^-1^ , related to the primary amine, were converted to one absorption band at a similar frequency. This change in FTIR results was considered as a spectroscopic marker to follow the reaction progress. After disappearance of the 2 bands of primary amine in the FTIR, the unreactive isophorone diisocyanate was evaporated by vacuum distillation at 160 °C. Next, the reaction mixture was allowed to cool at room temperature and then added to DEG-DDSQ (1.8 g, 1.23 mmol) solution in DMF (30 mL). The reaction mixture was heated within 10 min up to 45 °C under an argon atmosphere, and then stirred and refluxed for a further 28 h. The solvent was evaporated. Tpy-DDSQ was purified on a silica column using THF and hexane (1:1) as the eluent. Yield: 0.75 g, 41%.

**Figure 1:**
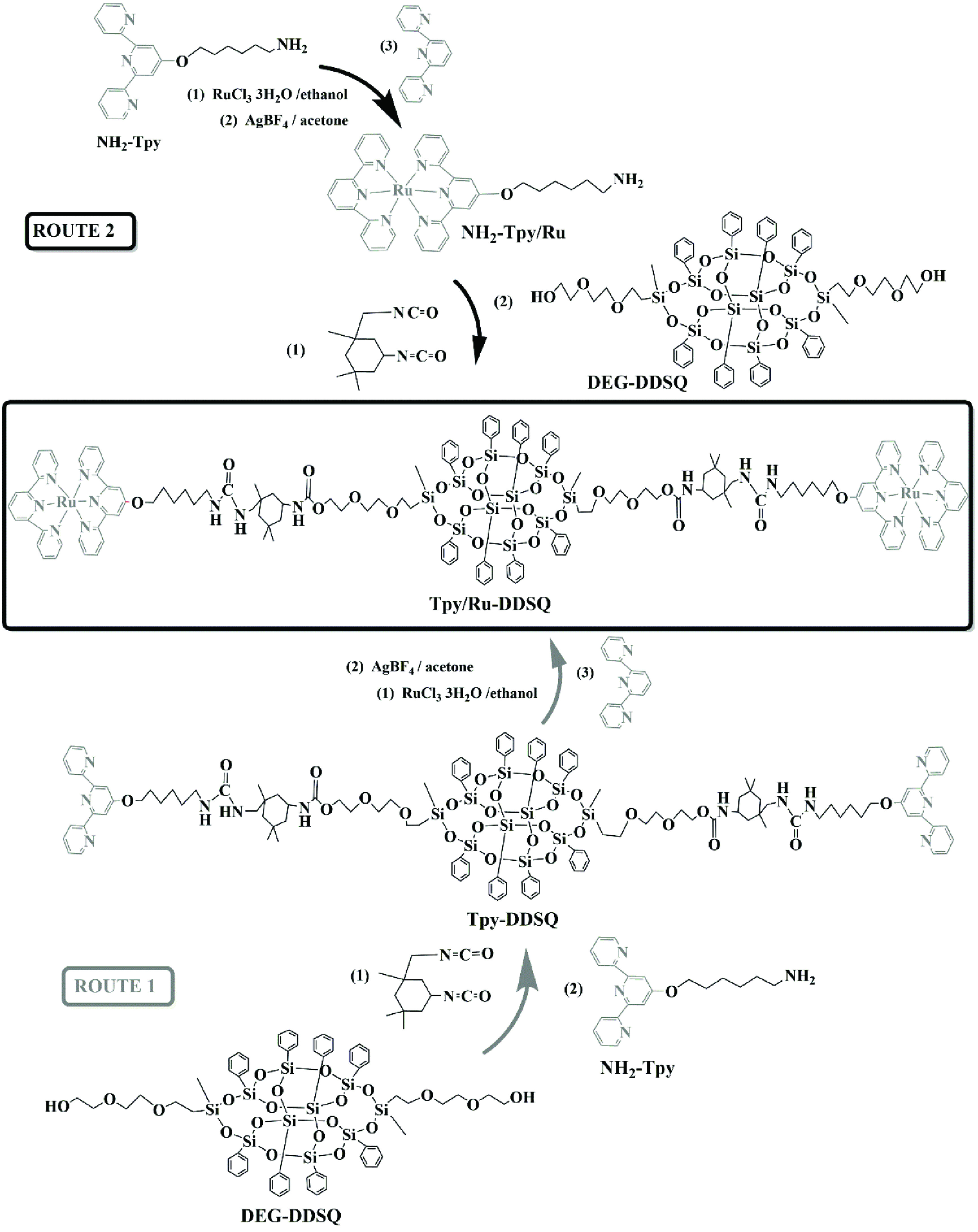
Synthesis of DDSQ-based Ru(II)-Tpy complex through 2 different approaches (Routes 1 and 2).

The calculated mass of Tpy-DDSQ was 2556.9. Therefore, the observed mass at m/z = 2663.9 [M + Ag]^+^ can be associated with the molecular ion peak of Tpy-DDSQ. Analyzing the mass of analyte was done in the presence of 1,8,9-anthracenetriol (dithranol) as a matrix and AgTFA as a salt. Therefore, it was believed that one of the terpyridine ligands formed a complex with the Ag ion. Results from MALDI-TOF/MS and ^1^H NMR showed that a reaction had occurred (Figure 1, Route 1).

Tpy-DDSQ: FTIR (cm^-1^) , ν = 3351 (N–H), ν = 3070-3020 (aromatic C–H), ν = 2960–2850 (aliphatic C–H), ν = 1644, 1606, 1567 (Tpy aromatic H), ν = 1135–1050 (Si–O and Si–C). ^1^H NMR (ppm) (CDCl_3_ , 400 MHz): d (ppm) δ_H_ 8.69 (2H, d, Tpy6-6”), 8.60 (2H, d, Tpy3,3”), 8.00 (2H, s, Tpy3’,5’), 7.84 (2H, t, Tpy4,4”), 7.36 (2H, t, Tpy5,5”), 7.54–7.18 (40H, phenyl protons on DDSQ, m), 4.22 (2H(f), m), 3.77–2.89 (14H(s,r,p,o,n,m,g), m), 2.29 (2H(a), m), 1.88–0.90 (21H(e,d,c,b,k,l,j,h,I,t), m), and 0.37 (6H(u), m).

#### 2.3.3. Complex formation, Tpy/Ru-DDSQ

The complexation reaction was performed according to procedures in the literature [20]. Briefly, a 2-fold excess amount of RuCl_3_ .3H_2_O (0.152 g, 0.58 mmol) in ethanol (40 mL) was added to the above solution of Tpy-DDSQ (0.75 g; 0.29 mmol) dissolved in DMF (150 mL). The mixture was stirred overnight at 110 °C under an argon atmosphere. Silver tetrafluoroborate (AgBF_4_ ; 0.34 g, 1.74 mmol) dissolved in acetone (30 mL) was added, and reflux was continued for 5 h. Next, an excess amount of 2,2’:6’,2”-terpyridine (0.7 mmol) dissolved in DMF (100 mL) was added to the reaction mixture and reflux was continued for a further 18 h. Finally, AgBF_4_ (0.34 g, 1.74 mmol) dissolved in acetone (30 mL) was added and refluxed for 24 h. The reaction mixture was allowed to cool at room temperature and the silver chloride was removed by filtration. The filtrate was concentrated, and after the addition of water, a precipitate appeared. The precipitate was isolated though centrifugation and was washed with water and dried in a vacuum overnight. Yield: 0.25 g, 33%.

Tpy/Ru-DDSQ: UV-Vis absorption spectrometer; absorption band at 280–300 nm ([π → π*]; transition for the alkynyl (phenyl groups attached to the DDSQ core) and Tpy ligand, absorption band at 400– 480 nm; metal-to-ligand charge-transfer (MLCT) for [Ru(tpy)_2_]^2+^ . ^1^H NMR (400 MHz, DMSO): d (ppm): 8.85 (2H, t, Tpy3’,5’), 8.59 (2H, m, Tpy3-3”), 7.79 (2H, s, Tpy4,4”), 7.30 (2H, m, Tpy6,6”), 7.28 (2H, t, Tpy5,5”), 4.20 (2H(f), m), 7.54–7.18 (40H, phenyl protons on DDSQ, m), 4.22 (2H(f), m), 3.77–2.89 (14H(s,r,p,o,n,m,g), m), 2.29 (2H(a), m), 1.88–0.90 (21H(e,d,c,b,k,l,j,h,I,t), m), and 0.37 (6H(u), m).

### 2.4. Synthesis of DDSQ-based Ru(II)-Tpy complex through Route 2

#### 2.4.1. Complex formation, NH_2_ -Tpy/Ru

The complexation reaction of Tpy-NH_2_ with RuCl_3_ .3H_2_O was prepared according to procedures in the literature [20]. Briefly, a solution of Tpy-NH_2_ (0.4 g, 1.15 mmol) in methanol (40 mL) was stirred at 110 °C. Next, an equimolar amount of RuCl_3_ .3H_2_O (0.3 g, 1.15 mmol) was added. Stirring was continued overnight. Silver tetrafluoroborate (0.68 g, 3.45 mmol) dissolved in acetone (30 mL) was added, and reflux was continued for 5 h. At the same temperature, an equimolar amount of 2,2’:6’,2”-terpyridine (0.27 g, 1.15 mmol) was added to the solution mixture and reflux was continued for a further 2^1^H. Finally, AgBF_4_ (0.68 g, 3.45 mmol) dissolved in acetone (30 mL) was added and refluxed for 24 h. The resulting dark orange precipitate was collected by filtration and washed twice with ice-cold water, followed by ethyl ether, and dried in a vacuum overnight. Yield: 0.20 g, 50%.

NH_2_ -Tpy/Ru: UV-Vis absorption spectrometer; [π → π*] transition of the Tpy ligand. 400–600 nm; MLCT for [Ru(Tpy)_2_]^2+^.

#### 2.4.2. Functionalizing of DDSQ with Ru(II) terpyridine complex, Tpy/Ru-DDSQ

A suspension of NH_2_ -Tpy/Ru (0.184 mmol) complex dissolved in DMF (50 mL) and an excess amount of isophorone diisocyanate (0.220 mmol) were refluxed together under argon atmosphere at 45 °C for 4 h. Progress of the reaction was followed by FTIR. After disappearance of the 2 bands of primary amine in the FTIR, the unreactive isophorone diisocyanate was evaporated by vacuum distillation at 160 °C. The reaction mixture was allowed to cool at room temperature. Next, 0.5 g (0.367 mmol) of DEG-DDSQ (2 equivalents of NH_2_ -Tpy) dissolved in DMF (50 mL) was added and refluxed for ^1^H. Next, the reaction mixture was washed with hexane and decanted. The black precipitate was isolated by filtration and dried in a vacuum overnight. Yield: 0.20 g, 40%.

Tpy/Ru-DDSQ: ^1^H NMR (ppm) (DMSO, 400 MHz): δH 9.08 (2H, t, Tpy3’,5’), 8.87 (2H, m, Tpy3-3”), 8.79 (2H, s, Tpy4,4”), 8.02 (2H, m, Tpy6,6”), 7.54–7.22 (2H, t, Tpy5,5”) and 40H, phenyl protons on DDSQ, m, 4.57 (2H(n), m), 4.00–2.52 (14H(s,r,p,o,n,m,g), m), 2.02 (2H(a), m), 1.81–0.76 (21H(e,d,c,b,k,l,j,h,i,t), m), and 0.37 (6H(u, m)). UV-Vis spectroscopy; absorption band at 280–300 nm ([π → π*]; transition for alkynyl (phenyl groups attached to the DDSQ core) and Tpy ligand, absorption band at 400–480 nm; MLCT for [Ru(tpy)_2_]^2+^. Section 2.1. gives the purity and source of all of the employed materials, as well as details of the instruments used.

## 3. Results and discussion

DDSQ substituted with 2 di(ethylene) glycol units (DEG-DDSQ), which was introduced recently [21–24], was used as an initial compound in the current research. In the case of Route 1, first, amine functionalized terpyridine (NH_2_ -Tpy; 6-aminohexyl 4’-(2,2’:6’,2”-terpyridine) ether) was synthesized as reported in the literature [20] with high yield (95%). Next, DEG-DDSQ was functionalized with NH_2_ -Tpy using an isocyanate agent. The yield of Tpy-DDSQ was 41% (Figure 1). The obtained compounds were characterized using FTIR, UV-Vis, ^1^H NMR, and MALDI-TOF/MS.

**Figure 2:**
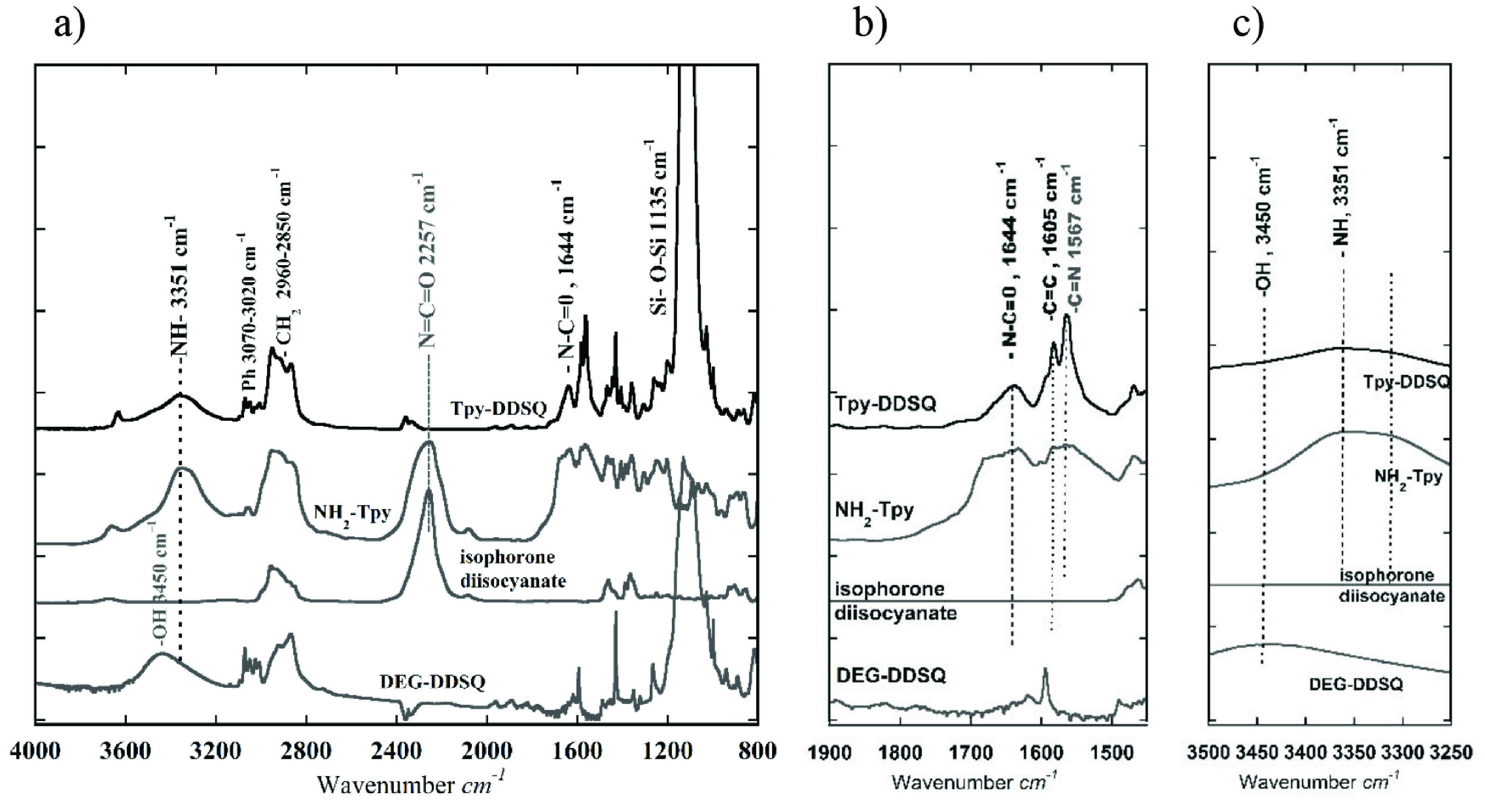
FTIR spectra of DEG-DDSQ, isophorone diisocyanate, NH_2_ -Tpy, and Tpy-DDSQ obtained from Route 1: (a) in the 800–4000 cm^-1^ region, (b) in the 1450–1900 cm^-1^ region, and (c) in the 3250–3500 cm^-1^ region.

Figure 2 portrays the comparison between the FTIR spectra of DEG-DDSQ, isophorone diisocyanate, NH_2_ -Tpy, and Tpy-DDSQ. In the FTIR spectrum of Tpy-DDSQ, the appearance of Si-O-Si absorbance at 1135 cm^-1^ indicated that the DDSQ core was preserved in the structure (Figure 2a). The appearance of C=C and C=N absorbances, respectively at 1605 cm^-1^ and 1567 cm^-1^ , indicated that aromatic rings belonging to Tpy were included in the DDSQ structure (Figure 2b). On the other hand, the OH stretching at 3450 cm^-1^ no longer existed, and at 3351 cm^-1^ , stretching related to the NH bond had newly appeared (Figure 2c). Particularly, these 2 differences in FTIR showed that a reaction took place between the OH group at the end of the DEG units and the –N=C=O groups in the isocyanate compound. All of these peaks showed successful attaching of the Tpy ligand to the DDSQ core.

**Figure 3:**
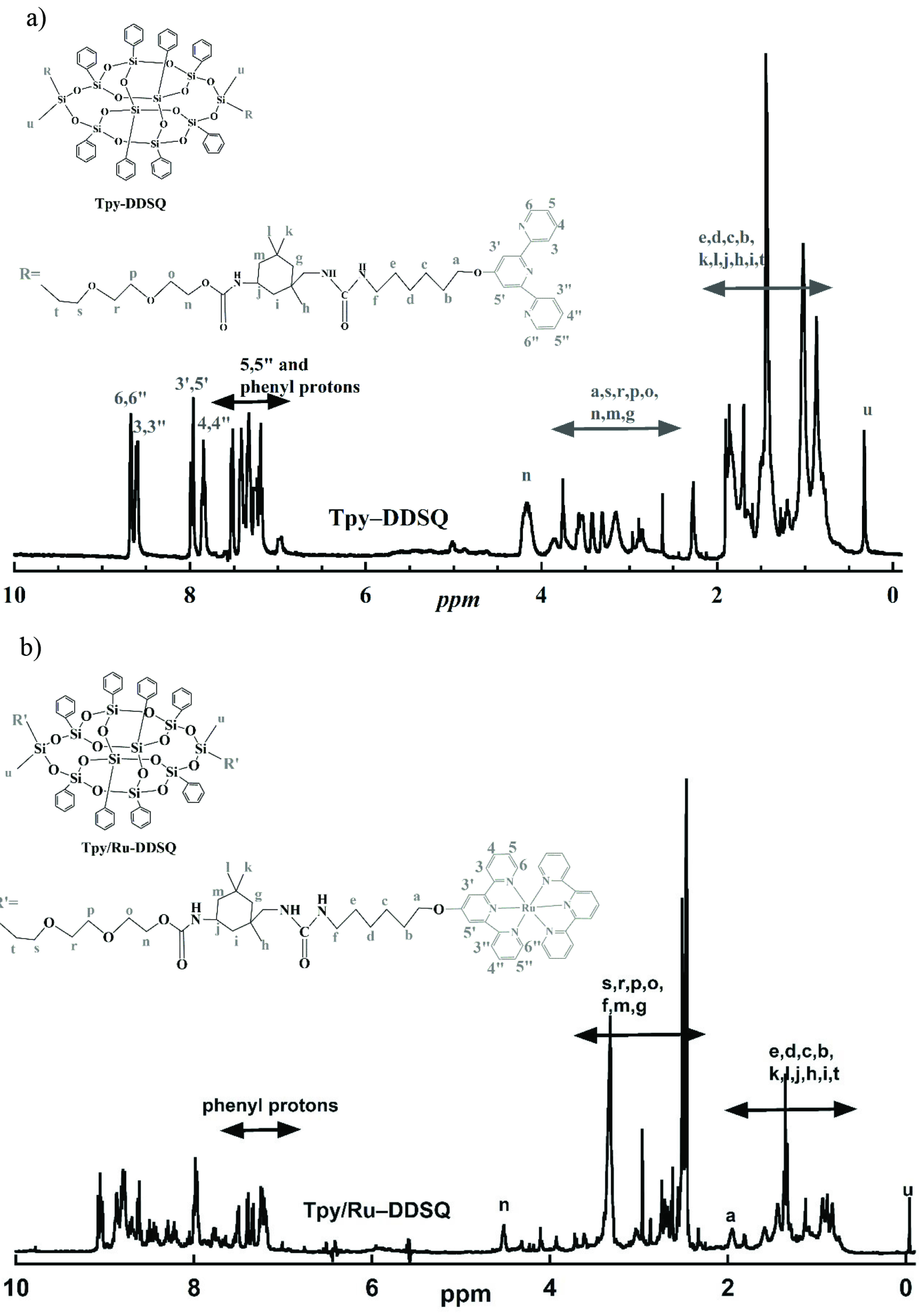
^1^H NMR spectra of Tpy-DDSQ in CHCl3 (a) and Tpy/Ru-DDSQ in DMSO (b) obtained from Route 1.

Further characterization was performed by ^1^H NMR spectroscopy (Figures 3a and 3b). Resonance of the aromatic and methyl protons belonging to the NH_2_ -Tpy and resonances of phenyl and di(ethylene) glycol protons belonging to DEG-DDSQ were clearly visible in the NMR spectrum of Tpy-DDSQ (Figure 3a). Each peak of Tpy-DDSQ was in accordance with the literature [20] and appeared in almost the same ranges without any chemical shifting (Figure 3a). These results showed that the Tpy ligands were successfully attached to the DDSQ core.

**Figure 4:**
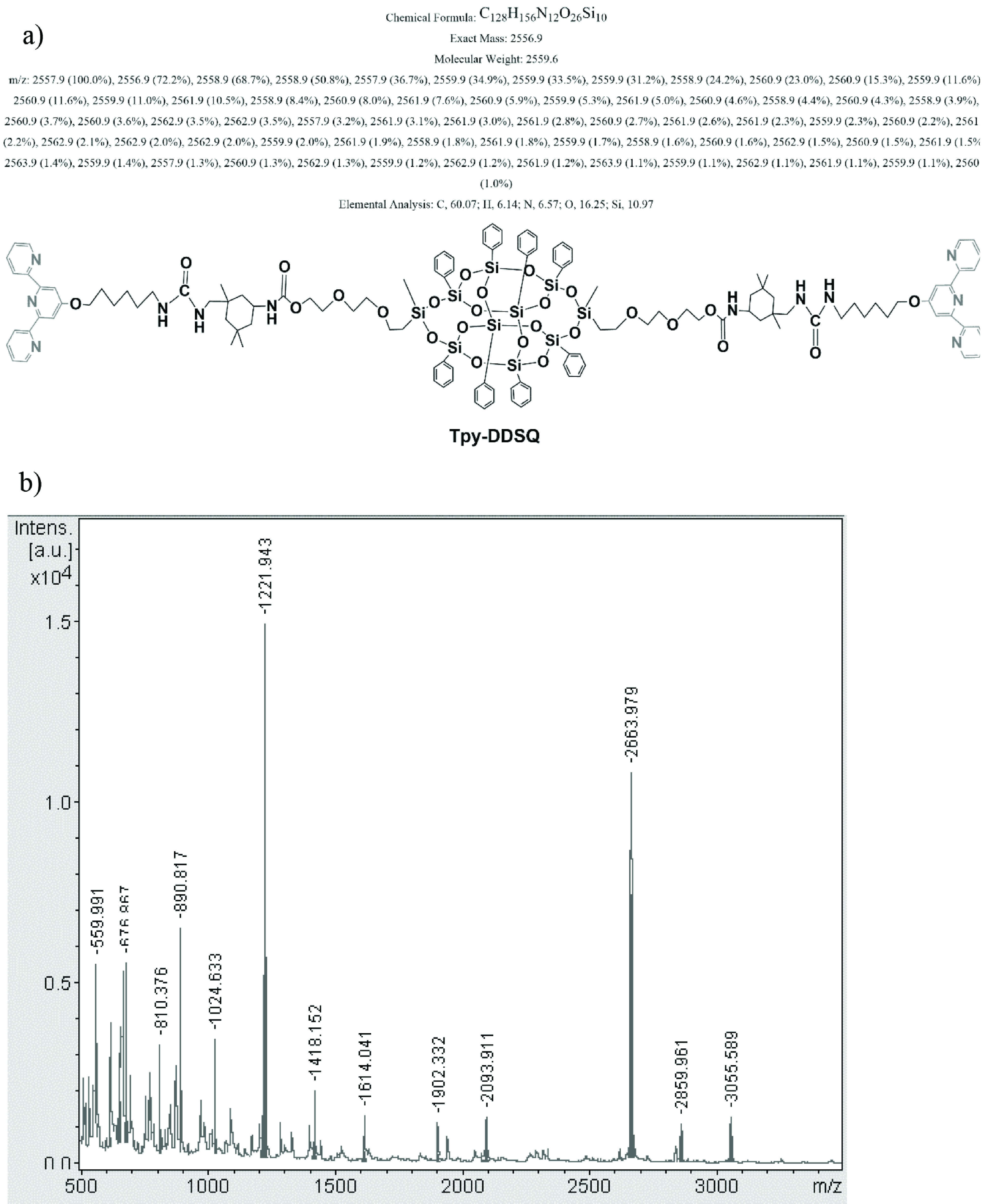
Structure of Tpy-DDSQ obtained from Route 1 (a) and MALDI-TOF/MS spectrum of Tpy-DDSQ (b).

In order to provide more precise information about the molar mass and structure of the synthesized Tpy- DDSQ, MALDI-TOF/MS results are presented in Figure 4. The theoretical molecular weight of Tpy-DDSQ is 2556.9 (Figure 4a). The base peak at m/z = 2663.9 [M + Ag]^+^ was associated with the molecular ion peak of Tpy-DDSQ (Figure 4b). As seen, the ^1^H NMR and MALDI-TOF/MS results were consistent with each other and confirmed the same structure of mono functional amino Tpy-DDSQ.

Finally, the complexation procedure was performed with transition metal ions of RuCl_3_ .3H_2_O in the presence of excess Tpy. The complexation yield was 33%. In Route 1, where the complexation took place in the last step, the yield of the DDSQ-based Ru(II)-Tpy complex was very low. As can be seen in the NMR results (Figure 3b) of the Tpy/Ru-DDSQ molecule obtained from Route 1, complexation was not fully established. The excess peaks in the range of 7–9 ppm can be explained by partial complexation of Tpy-DDSQ (i.e. mono complexation of some Tpy-DDSQ molecules). Experimentally, only 25% of the DDSQ used in the complexation reaction was able to be converted to the Tpy/Ru-DDSQ complex.

**Figure 5:**
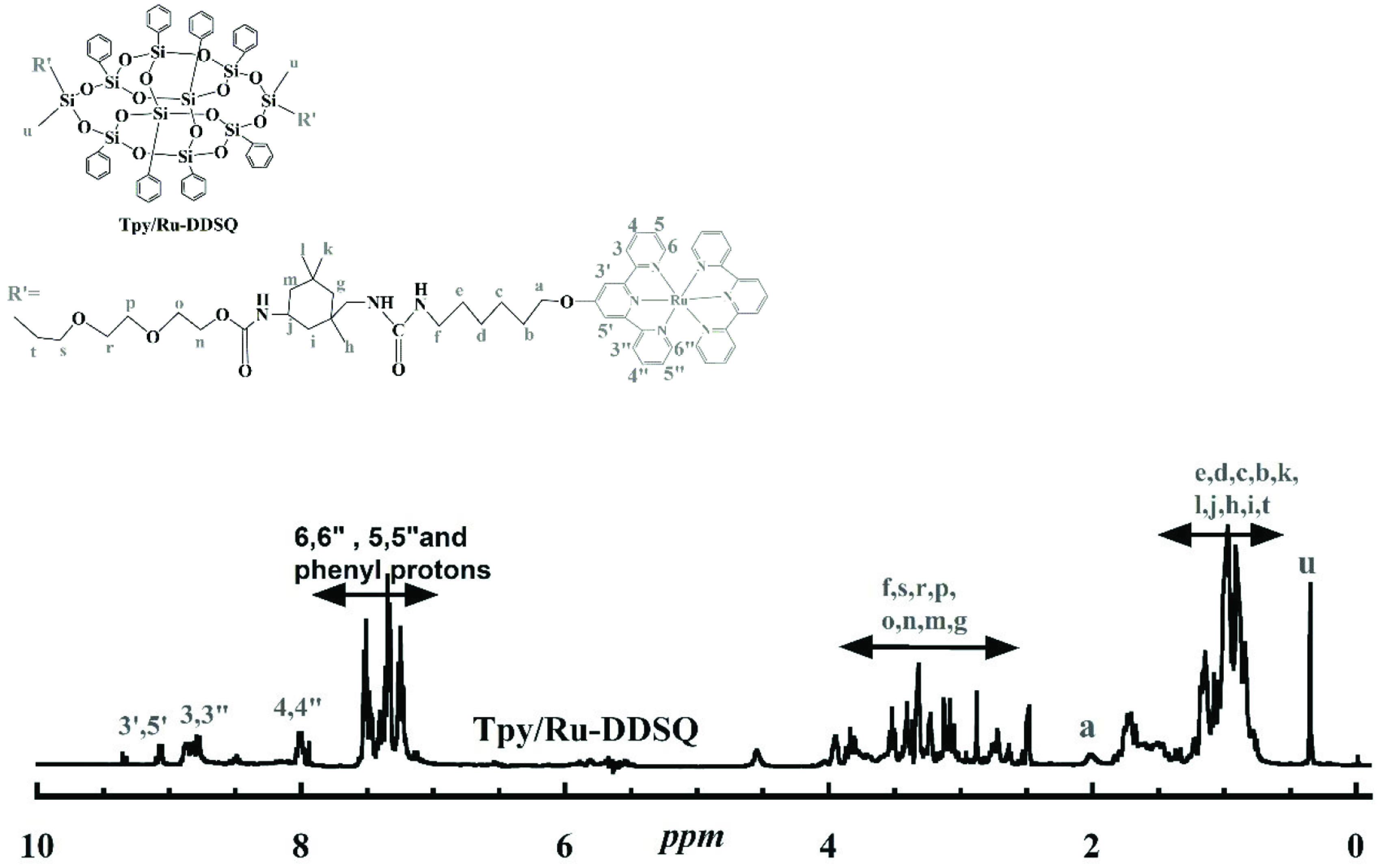
^1^H NMR spectrum of Tpy/Ru-DDSQ obtained from Route 2.

The first step of Route 2 was the complexation of NH_2_ -Tpy with transition metal ions of RuCl_3_ .3H_2_O in the presence of Tpy, which resulted in NH_2_ -Tpy/Ru with a yield of 50% (Figure 1). Next, NH_2_ -Tpy/Ru was attached to the DDSQ core using an isocyanate agent and resulted in the DDSQ-based Ru(II)-Tpy complex (Tpy/Ru-DDSQ) with a yield of 37%. The obtained complex of Tpy/Ru-DDSQ was characterized by ^1^H NMR and XPS spectroscopy measurements. The ^1^H NMR spectrum of Tpy/Ru-DDSQ is portrayed in Figure 5. As in previous reports [18–20], after the complexation reaction, the protons in positions 3’,5’ and 6,6” were shifted, respectively, to lower and higher fields due to the conformational change from the antiperiplanar to synperiplanar. On the other hand, the aromatic protons of Tpy in positions 5,5” and 6,6” overlapped with those of the phenyl groups attached to the DDSQ core (Figure 5). All of the peaks were in agreement with those given in the literature [18–20] and indicated that the Tpy/Ru-DDSQ complex was obtained with the same structure as that obtained from Route 1. On the other hand, this NMR was clearer and the peaks were more understandable when compared to the final NMR belonging to Tpy/Ru-DDSQ obtained from Route 1.

**Figure 6:**
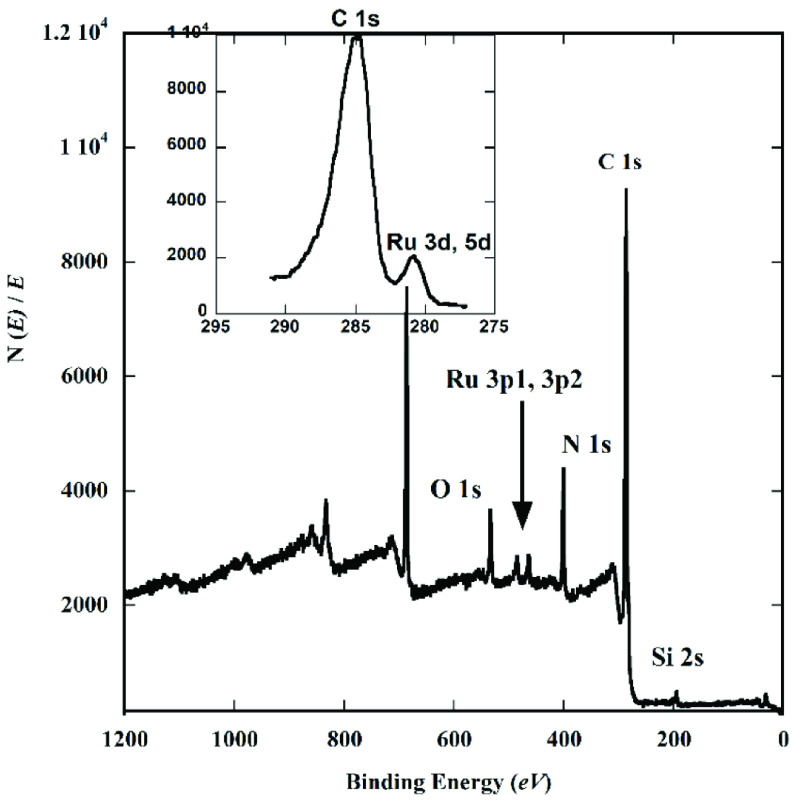
XPS spectrum of Tpy/Ru-DDSQ complex obtained from Route 2.

The composition of the complex compound of Tpy/Ru-DDSQ was examined by XPS. The XPS spectrum shows peaks at 534, 400, 288, 155, 102, and 281 eV, which, respectively, indicated the presence of oxygen (1s), nitrogen (1s), carbon (1s), silicon (2s), silicon (1s), and ruthenium (3d) (Figure 6). The presence of the Ru signal in the XPS spectrum confirmed the expected complex compound of Tpy/Ru-DDSQ.

In Route 2, the yield of the functionalization process of DDSQ with the Ru(II)-Tpy complex was 40%. Since functionalization occurs after complexation, the value of 40% indicated the converting ratio of DDSQ to Tpy/Ru-DDSQ complex. This yield was promising when compared to that obtained in Route 1 (25%).

**Figure 7:**
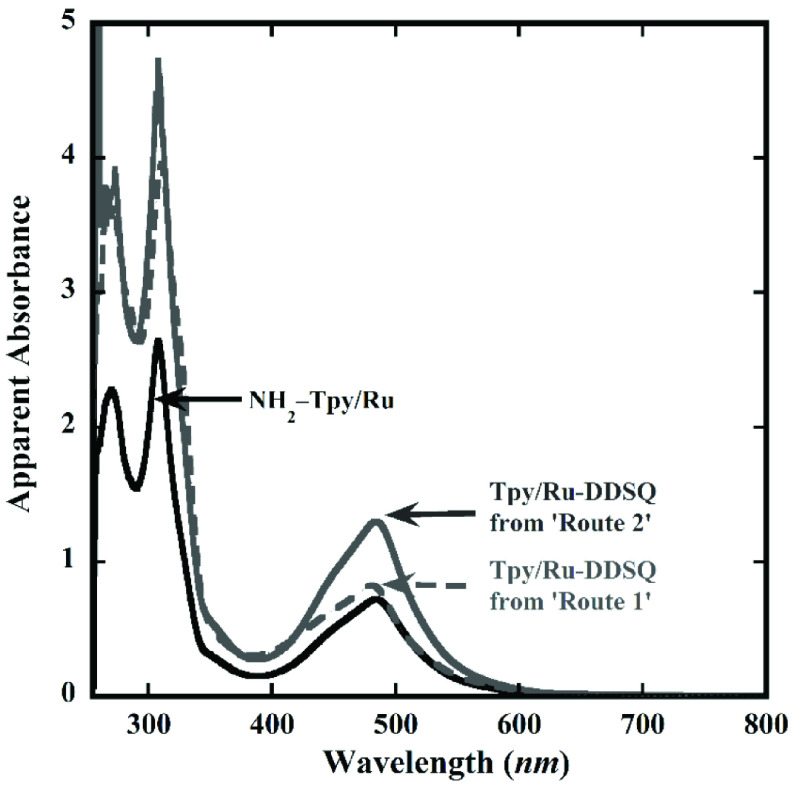
UV-Vis absorption spectra of Tpy/Ru-DDSQ obtained from Route 1 (dotted and red line) and Route 2 (solid and blue line), and NH_2_ -Tpy/Ru (solid and gray line). The concentration of all of the samples was 3.3 10−5 M in acetone.

The optical properties of the Tpy/Ru-DDSQ complexes obtained through Routes 1 and 2 were examined separately by UV-Vis spectroscopy. The reddish solution of Tpy/Ru-DDSQ in acetone showed high- and lowenergy absorption bands, as seen in Figure 7. The high-energy absorption band at 280–300 nm was assigned to the intraligand [π → π*] transition of phenyl groups attached to the DDSQ core and Tpy ligand, while the low-energy absorption band at 400–480 nm was assigned to the MLCT for [Ru(Tpy)_2_]^2+^ , which was compatible with the literature [18–20].

A model compound (NH_2_ -Tpy/Ru complex) consisting of [Ru(Tpy)_2_]^2+^ moiety was formed. The concentration of the Tpy/Ru-DDSQ complexes obtained from Routes 1 and 2 were the same as that of the model complex, NH_2_ -Tpy/Ru, which was 3.3 10−5 M. Since complexes NH_2_ -Tpy/Ru and Tpy/Ru-DDSQ had the same optical compounds, their absorbance characteristic and intensity should be the same. Theoretically, Tpy/Ru-DDSQ consists of 2 units of [Ru(Tpy)_2_]^2+^ moieties in one structure. Therefore, its absorbance intensity at high- and low-energy absorbance bands should be 2 times higher than that of NH_2_ -Tpy/Ru. Although the high-energy absorption bands of DDSQ obtained from Routes 1 and 2 were almost the same, the MLCT of Tpy/Ru-DDSQ obtained from Route 1 was lower than that obtained from Route 2 (Figure 7). The lack of some [Ru(Tpy)_2_]^2+^ moieties means lower MLCT. Therefore, partial complexation of Tpy-DDSQ molecules should be assumed first. In the NMR for Tpy/Ru-DDSQ obtained from Route 1, the excess peaks in the range of 7–9 ppm, which were attributed to partial complexation of Tpy-DDSQ (via 1 arm), were in agreement with this assumption. Moreover, according to the UV-Vis spectroscopy, the absence of an absorption band around 400 nm indicated that half of the complex of Ru(III) had not formed. It can be assumed that complete complexation with Ru(III) took place in both Routes 1 and 2. Thus, partial complexation of Tpy-DDSQ molecules can be considered as the main reason for the low MLCT of Route 1.

The limitation of Route 1 was also confirmed in a study by Kucuk et al. [20]. According to the UV-Vis spectroscopy results, only 22% of the Ru(II)-Tpy moieties were included in the DDSQ nanostructured blocks, where complexation occurred in the last step. The conversion ratio of Route 1 (25%) obtained in this study was consistent with the abovementioned study results.

In conclusion, two different approaches (Routes 1 and 2) have been discussed in the synthesis of a DDSQ-based metallo-supramolecular complex (Tpy/Ru-DDSQ). In Route 1, complexation was followed by the functionalization of DDSQ with a Tpy ligand, whereas in Route 2, complexation of Tpy was performed prior to binding of Tpy to DDSQ. The yields of complexation in Routes 1 and 2 were similar. However, the incorporation ratio of DDSQ into the metallo-supramolecular complex was 40% in Route 2, while it was only 25% in Route 1. On the other hand, the NMR analyses clearly showed that Route 2 provided a purer DDSQ-based complex structure (Tpy/Ru-DDSQ). In addition, the UV-Vis spectrum demonstrated that in order to benefit more from the DDSQ structure, it is essential to follow Route 2, in which complexation is the first priority,

## References

[1] (2014). A trilayer film approach to multicolor electrochromism. Journal of the American Chemical Society.

[2] (2015). -di(1H-pyrazol-1-yl)pyridine substituted cyclotri- and polyphosphazene ruthenium(II) complexes: Chemical and physical behaviour. Terpyridine and 2.

[3] (2005). Improved performance of OLEDs with ITO surface treatments. Thin Solid Films.

[4] (2005). Luminescence enhancement of ruthenium complexes in polymer nanosheet by surface plasmon resonance of metal nanoparticle. Chemistry Letters.

[5] (2002). Photocurrent enhancement for polymer Langmuir-Blodgett monolayers containing ruthenium complex by surface plasmon resonance. ýJournal of Physical Chemistry B.

[6] (1980). Polymer-films on electrodes. 4. Nafion-coated electrodes and electrogenerated chemi-luminescence of surface-attached Ru(Bpy)32$+$. Journal of the American Chemical Society.

[7] (2009). Synthesis, characterization and thermal properties of a novel star polymer consisting of poly(epsilon-caprolactone) arms emanating from an octa-functional porphyrazine core. Reactive and Functional Polymers.

[8] (2012). Star poly(N-isopropylacrylamide) tethered to polyhedral oligomeric silsesquioxane (POSS) nanoparticles by a combination of ATRP and click chemistry. Journal of Nanomaterials.

[9] (2003). Synthesis and characterization of fillers of controlled structure based on polyhedral oligomeric silsesquioxane cages and their use in reinforcing siloxane elastomers. Journal of Polymer Science Part B.

[10] (2007). and self-cleaning coatings. Polymers for Advanced Technologies.

[11] (2018). Ion conducting behavior of silsesquioxane-based materials used in fuel cell and rechargeable battery applications. Journal of Structural Chemistry.

[12] Proton-conducting membrane based on newly synthesized silsesquioxane derivatives. Japan Patent Office 2015; Japan.

[13] (2004). Polymeric nanocomposites - Polyhedral oligomeric silsesquioxanes (POSS) as hybrid nanofiller. Journal of Macromolecular Science-Polymer Reviews.

[14] (2005). Structure, dynamic properties, and surface behavior of nanostructured ionomeric polyurethanes from reactive polyhedral oligomeric silsesquioxanes. Macromolecules.

[15] (1996). Hybrid organic-inorganic thermoplastics: Styryl-based polyhedral oligomeric silsesquioxane polymers. Macromolecules.

[16] (2001). Designed hybrid organic-inorganic nanocomposites from functional nanobuilding blocks. Chemistry of Materials.

[17] (2017). Supramolecular assembly of platinum-containing polyhedral oligomeric silsesquioxanes: An interplay of intermolecular interactions and a correlation between structural modifications and morphological transformations. Chemical Science.

[18] (2014). Metallo-supramolecular materials based on terpyridine-functionalized polyhedral silsesquioxane. Polymer International.

[19] (2000). New metallodendrimers containing an octakis(diphenylphosphino)-functionalized silsesquioxane core and ruthenium(II)-based chromophores. Inorganic Chemistry.

[20] (2018). Synthesis and photochemical response of Ru(II)-coordinated double-decker silsesquioxane. RSC Advances.

[21] (2011). Effects of hydrogen bonding on the monolayer properties of amphiphilic double-decker-shaped polyhedral silsesquioxanes. Langmuir.

[22] (2011). Langmuir-Blodgett films composed of amphiphilic double-decker shaped polyhedral oligomeric silsesquioxanes. Journal of Colloid and Interface Science.

[23] (2012). Proton-conducting electrolyte film of double-decker-shaped polyhedral silsesquioxane containing covalently bonded phosphonic acid groups. Journal of Materials Chemistry A.

[24] (2013). Effects of subphase composition on the monolayer behavior of ``core-coronae'' type hybrid amphiphiles. Thin Solid Films.

